# Unexpected Transformations during Pyrroloiminoquinone Biosynthesis

**DOI:** 10.1021/jacs.4c03677

**Published:** 2024-05-08

**Authors:** Josseline Ramos Figueroa, Lingyang Zhu, Wilfred A. van der Donk

**Affiliations:** Department of Chemistry and Howard Hughes Medical Institute, University of Illinois at Urbana−Champaign, Urbana, Illinois 61801, United States

## Abstract

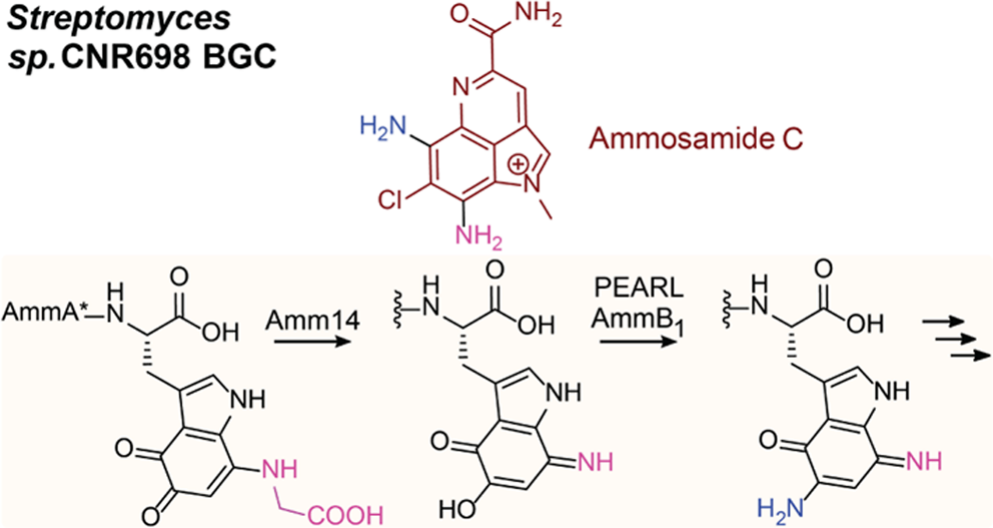

Pyrroloiminoquinone-containing natural products have long been known for their biological activities. They are derived from tryptophan, but their biosynthetic pathways have remained elusive. Studies on the biosynthetic gene cluster (BGC) that produces the ammosamides revealed that the first step is attachment of Trp to the C-terminus of a scaffold peptide in an ATP- and tRNA-dependent manner catalyzed by a PEptide Aminoacyl-tRNA Ligase (PEARL). The indole of Trp is then oxidized to a hydroxyquinone. We previously proposed a chemically plausible and streamlined pathway for converting this intermediate to the ammosamides using additional enzymes encoded in the BGC. In this study, we report the activity of four additional enzymes from two gene clusters, which show that the previously proposed pathway is incorrect and that Nature’s route toward pyrroloiminoquinones is much more complicated. We demonstrate that, surprisingly, amino groups in pyrroloiminoquinones are derived from (at least) three different sources, glycine, asparagine, and leucine, all introduced in a tRNA-dependent manner. We also show that an FAD-dependent putative glycine oxidase (Amm14) is required for the process that incorporates the nitrogens from glycine and leucine and that a quinone reductase is required for the incorporation of asparagine. Additionally, we provide the first insights into the evolutionary origin of the PEARLs as well as related enzymes, such as the glutamyl-tRNA-dependent dehydratases involved in the biosynthesis of lanthipeptides and thiopeptides. These enzymes appear to all have descended from the ATP-GRASP protein family.

## Introduction

**Figure 1:**
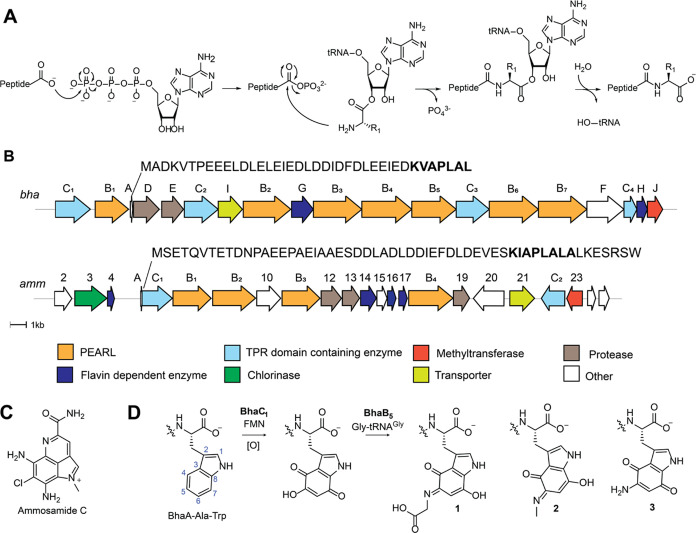
Biosynthesis of pyrroloiminoquinones and related compounds. (A) General reaction scheme for peptide aminoacyl-tRNA ligases (PEARLs). (B) Two related BGCs that are thought to result in pyrroloiminoquinone products. The *amm* BGC has been demonstrated to generate ammosamide C; the product of the *bha* BGC is currently not known. (C) Structure of ammosamide C. (D) After addition of an Ala and Trp at the C-terminus of the precursor peptide BhaA, BhaC_1_ oxidizes the indole of the Trp, and the PEARL BhaB_5_ adds Gly from Gly-tRNA^Gly^, resulting in three observed products during heterologous production in *E. coli*. Similar reactions are catalyzed by AmmC_1_ and AmmB_3_ on their precursor peptides. A previous study suggested that compound **1** was off-pathway by premature dissociation from the respective PEARLs, whereas compound **2** was proposed to be on-pathway to compound **3**. The current study indicates that compound **1** is on-pathway to compound **3** and that compound **2** is off-pathway.

Peptide aminoacyl-tRNA ligases (PEARLs) catalyze the appendage of amino acids to the C-terminus of scaffold peptides in an ATP- and aminoacyl-tRNA-dependent manner.^1^ ATP is used to phosphorylate the C-terminus of their peptide substrates to form an acylphosphate intermediate, which is attacked by the amine group of an aminoacyl-tRNA to generate a new peptide bond (Figure 1A).^2^ Subsequent hydrolysis of the tRNA results in the net addition of an amino acid to the peptide. These enzymes have been shown to add a wide variety of amino acids to the C-termini of their peptide substrates. The first examples involved the installation of cysteine residues to a precursor peptide en route to the biosynthesis of 3-thiaglutamate and related molecules.^1−3^ More recently, PEARL enzymes that are involved in the biosynthesis of pyrroloiminoquinone-derived natural products were characterized.^4^ These compounds (also termed pyrroloquinolines) include the ammosamides (e.g., Figure 1C) produced by the marine organism *Streptomyces* sp. CNR-698 and by *Streptomyces uncialis* DCA2648,^5−7^ which display an array of different bioactivities.^8−14^

Previous investigation of pyrroloiminoquinone biosynthesis focused on two biosynthetic gene clusters (BGCs, Figure 1B) that share a highly similar precursor peptide, the ammosamide BGC (*amm*) and a gene cluster in *Bacillus halodurans* C-125 (*bha*). The final product for the latter is not currently known as it is cryptic under the conditions tested. The prior study used heterologous expression of biosynthetic enzymes in *Escherichia coli* to assign function to individual enzymes. The availability of two BGCs from different phyla was beneficial for assignment of function to orthologous shared enzymes as the *bha* enzymes generally provided higher product yields that allowed NMR characterization.^4^ Two PEARL enzymes from these BGCs (BhaB_7_ and AmmB_2_) were shown to append a Trp moiety to a precursor peptide that serves as the core of the pyrroloiminoquinones (Figure 1D). Trihydroxylation of the added Trp residue is catalyzed by an FMN-dependent oxidoreductase (BhaC_1_ or AmmC_1_). While the initial product of the flavoprotein is in the hydroquinone state, oxidation to the quinone form is needed to activate this biosynthetic intermediate for the next PEARL-catalyzed reaction. BhaB_5_ was shown to modify the vinylogous carboxylate at C5 in the oxidized Trp by attaching a glycine residue transferred from Gly-tRNA^Gly^ (Figure 1D).^4^

In the reaction catalyzed by BhaB_5_, a mixture of products was obtained. Based on LC-MS and NMR characterization, they were assigned as the tryptophanyl-derived glycine-quinone adduct (**1**), a decarboxylated product (**2**, or its tautomer), and an aminoquinone (**3**) (Figure 1D). We proposed that the glycine-quinone adduct **1** was an off-pathway intermediate formed by dissociation from the enzyme active site and that compounds **2** and **3** were on-pathway. The analogous reaction catalyzed by AmmB_3_ in *E. coli* resulted only in production of a peptide similar to **2** derived from the AmmA substrate. We hypothesized that the aminoquinone product **3** requires first oxidation of **2** by an oxidase present in *E. coli*, resulting in hydrolysis to release formaldehyde and formation of the amino group that is present in the final product ammosamide. We anticipated that in the producing organism, this oxidation would be catalyzed by one of the remaining enzymes in the BGC. We also suggested that the two remaining PEARLs in the ammosamide BGC (AmmB_1_ and AmmB_4_) would utilize Gly-tRNA^Gly^ to introduce the additional two amino groups that are present in the final product (Figures 1C and S1). We show herein that none of these hypotheses are correct and that the biosynthesis of ammosamide and related molecules is more complex than anticipated.

With the aim to find the next enzymatic steps in the biosynthetic pathways encoded by the two gene clusters described above, we investigated the activity of four additional enzymes that are encoded in the BGCs shown in Figure 1B. We show that the aforementioned aminoquinone **3** and the decarboxylated product (**2**) are nonenzymatic decomposition products of the unstable glycine-quinone adduct **1**. In the actual biosynthetic pathway, peptide **1** is modified by a predicted glycine oxidase encoded in both BGCs, forming the aminoquinone product **3** through glyoxylate release. Investigation of the remaining unassigned PEARL enzymes of both clusters revealed new PEARL-catalyzed transformations. In the *B. halodurans* C-125 pathway, after formation of aminoquinone **3**, an asparagine is installed at the C-terminus of the modified peptide in an Asn-tRNA^Asn^-dependent fashion. In the ammosamide pathway, after aminoquinone formation, leucine (and not glycine) was added to the aminoquinone, providing the second amino group present in the ammosamide core. These findings show that the biosynthetic logic that led to the evolution of the pathway toward ammosamide and related products is complex and that seemingly similar BGCs and precursor peptides have diverged in their pathways. Through bioinformatic and structural analysis, we provide new insights into the potential evolutionary origin of PEARLs, and by extension glutamyl-tRNA-dependent lanthipeptide and thiopeptide dehydratases.^15^

## Results and Discussion

### Glycine Oxidase BhaG Oxidizes the Glycine-Quinone Adduct **1**

**Figure 2:**
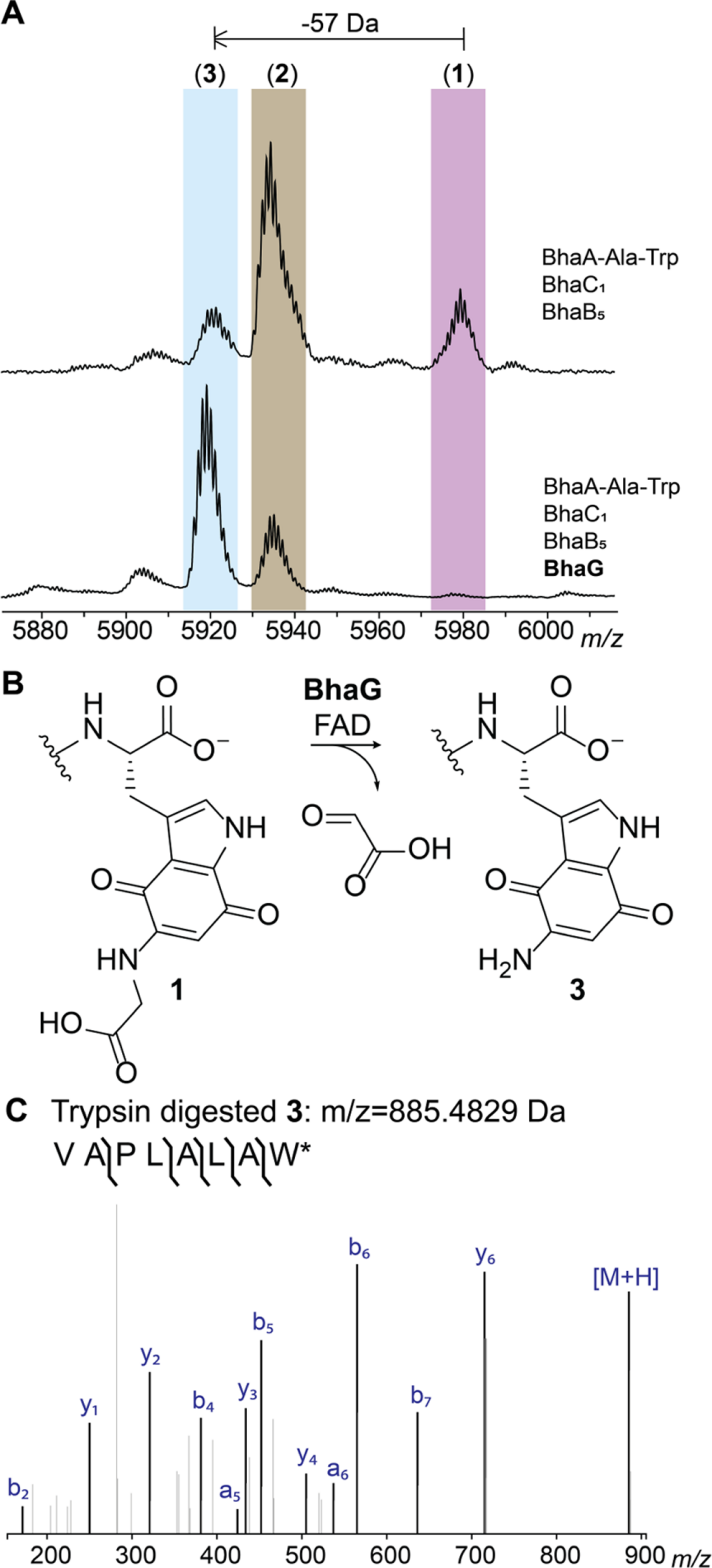
Activity of BhaG. (A) Coexpression of Bha-Ala-Trp with BhaC_1_ and BhaB_5_ in *E. coli* and subsequent MALDI-TOF MS analysis results in detection of the glycine adduct **1**, methyliminoquinone **2**, and aminoquinone **3**. Inclusion of BhaG in the coexpression experiment leads to disappearance of the glycine adduct **1**, strong reduction of the peak of peptide **2**, and formation of predominately **3**. Differences in ionization efficiency in MALDI-TOF MS underestimate the relative amount of product **3** (see LC-MS data in Figure S2). (B) Reaction catalyzed by BhaG. (C) ESI-MS/MS analysis of the C-terminal peptide of trypsin-digested product **3** formed in vitro by BhaG (calculated *m*/*z* = 885.4829, observed *m*/*z* = 885.4827). W* = aminoquinone derivative of Trp.

To uncover the next biosynthetic step in the *bha* pathway, the peptide BhaA-Ala-Trp was heterologously coexpressed in with the previously characterized modifying enzymes (BhaC_1_ and BhaB_5_; Figure 1D)^4,16^ as well as iterative inclusion of each of the remaining enzymes encoded in the *bha* cluster (Figure 1B). After peptide purification and desalting, the samples were analyzed by matrix-assisted laser desorption/ionization time-of-flight (MALDI-TOF) mass spectrometry (MS). Out of all individual coexpression experiments, only the inclusion of a putative glycine oxidase, BhaG (UniProt Q9KB87), resulted in production of a new peak with a decreased mass of 57 Da from the glycine adduct **1** (Figure 2A). We confirmed that the modification occurred at the C-terminal Trp using high-resolution MS/MS on a trypsin-generated fragment as shown in Figure 2C.

Analysis by liquid chromatography (LC) coupled with electrospray ionization (ESI) mass spectrometry showed that peptide **1** was the major product of BhaB_5_ in the absence of BhaG, with minor amounts of compounds **2** and **3** (Figure S2). This result suggested that **1** was not an off-pathway product, as previously hypothesized, but the actual product of the reaction catalyzed by BhaB_5_. To confirm that the glycine adduct **1** is converted by BhaG to aminoquinone **3**, the enzyme was purified and used for *in vitro* reactions. His_6_-BhaG copurified with FAD (Figure S3) in agreement with other glycine oxidases.^17^ Compounds **1**, **2**, and **3** coelute under our purification protocols and could not be separated. Therefore, the mixture was used for *in vitro* studies, which contained **1** as the major component (Figure S2). *In vitro* reconstitution of BhaG activity showed that the glycine adduct **1** was indeed converted into aminoquinone product **3** (Figure S4). The enzymatic conversion was expected to involve hydride transfer from the α-carbon of the glycine moiety to FAD, followed by hydration of the resulting imine to generate a tetrahedral intermediate that eliminates glyoxylate and forms **3** (Figure S5A). Hence, we investigated whether glyoxylate was formed over the course of the reaction by derivatization of the reaction products using phenylhydrazine (Figure S5B). As expected, the phenylhydrazone adduct of glyoxylate was formed as shown by coinjection with a standard mixture obtained from glyoxylic acid. Thus, the Gly adduct **1** is not converted to **3** by decarboxylation, oxidation, and hydrolysis to provide formaldehyde as previously proposed (Figure 1D)^4^ but instead by enzymatic oxidation by a glycine oxidase that is conserved in both BGCs.

### BhaB_4_ Is an Asn-tRNA^Asn^-Dependent PEARL

**Figure 3:**
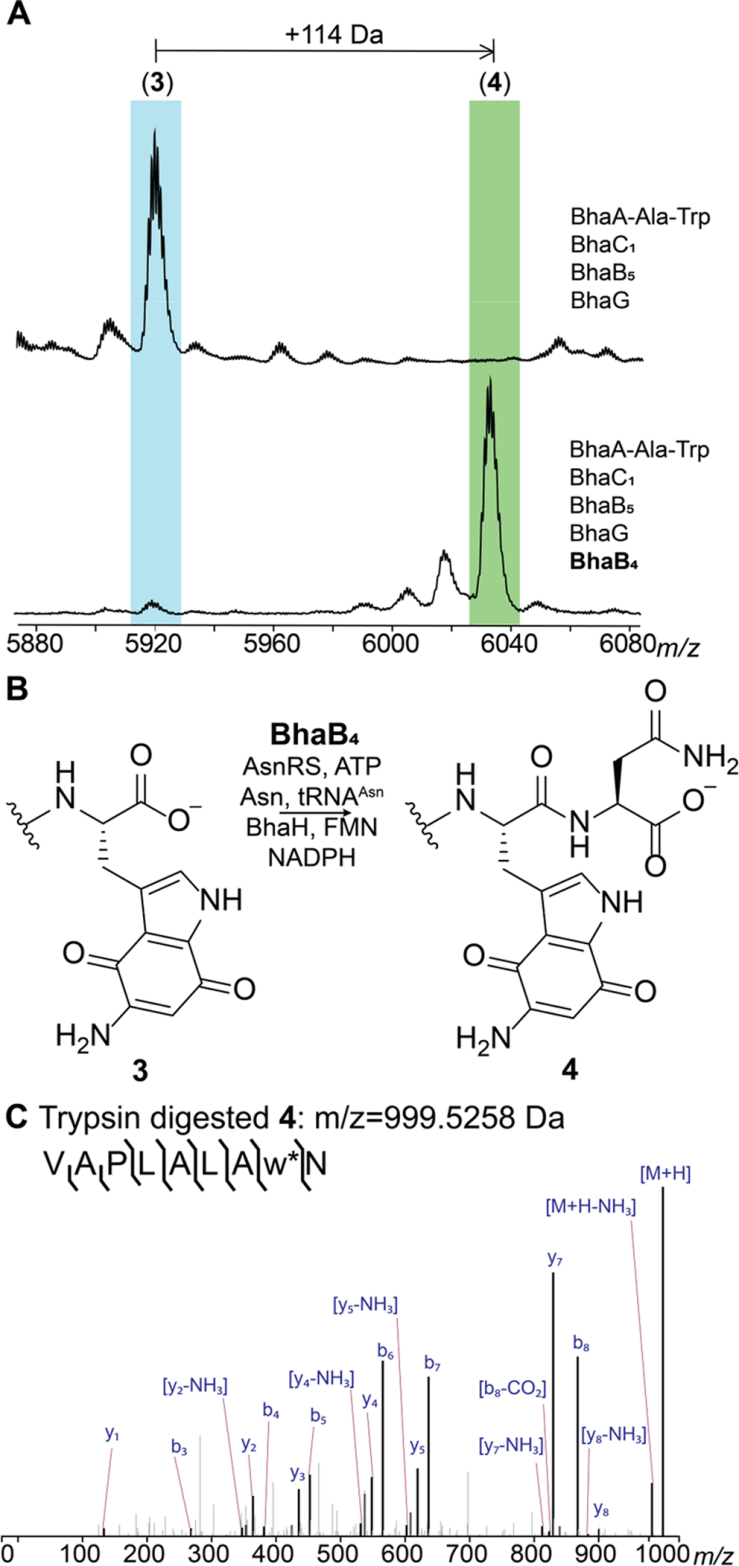
BhaB_4_ adds Asn to the C-terminus of intermediate **3**. (A) Inclusion of BhaB_4_ in the coexpression system that produces **3** results in the formation of a new product that is increased in mass by 114 Da. (B) The reaction catalyzed by BhaB_4_. (C) ESI-MS/MS analysis of the C-terminal fragment upon trypsin digestion of peptide **4** (calculated *m*/*z* = 999.5258, observed *m*/*z* = 999.5282; Figure S7B). w* = aminoquinone derivative of Trp.

In previous experiments, we were unable to identify the next PEARL to act in the pathway, which may be explained by the need to include BhaG to efficiently generate peptide **3**. Thus, we investigated whether this new information might allow the identification of the next biosynthetic step. We coexpressed BhaA-Ala-Trp, BhaC_1_, BhaB_5_, and BhaG and in turn added genes encoding each of the remaining PEARL enzymes to the expression system. As shown in Figure 3A, BhaB_4_ (UniProt Q9KB87) resulted in an increase in the peptide mass by 114 Da, whereas the addition of the other PEARLs did not yield any observable mass shift. High-resolution MS/MS analysis showed that the increased mass was generated by asparagine condensation to the C-terminal carboxylate of the modified tryptophan (Figure 3B,C). To confirm that asparagine was the amino acid added by BhaB_4_, as well as the site of the new amide bond formation, NMR analysis of the peptide was performed. Confirmation that the Asn residue was added to the C-terminus to produce peptide **4** was provided by a characteristic NOESY peak from the NH proton (δ 7.95 ppm) of the newly added Asn to the β protons (δ 3.10 and 2.87 ppm) of the modified Trp (Figure S6A).

Next, we investigated whether the activity of BhaB_4_ could be recapitulated *in vitro* using purified aminoquinone intermediate **3**. Initial attempts to detect the desired mass shift using tRNA^Asn^, Asn-tRNA^Asn^ transferase from *E. coli* (AsnRS), BhaB_4_, ATP, **3**, and Asn did not result in product formation. However, inclusion of an aliquot of *E. coli* cell free extract did result in formation of the +114 Da product (Figure S7). We hypothesized that an enzyme in the *E. coli* extract was modifying the redox state of aminoquinone **3**, which is obtained in the oxidized form in our purification protocols. This hypothesis was further fueled by the presence of a putative NADPH-dependent FMN-quinone reductase in the *bha* cluster (BhaH, UniProt Q9KB78, Figure 1A). Thus, we investigated whether the need for *E. coli* cell free extract could be obviated by inclusion of BhaH.

**Figure 4:**
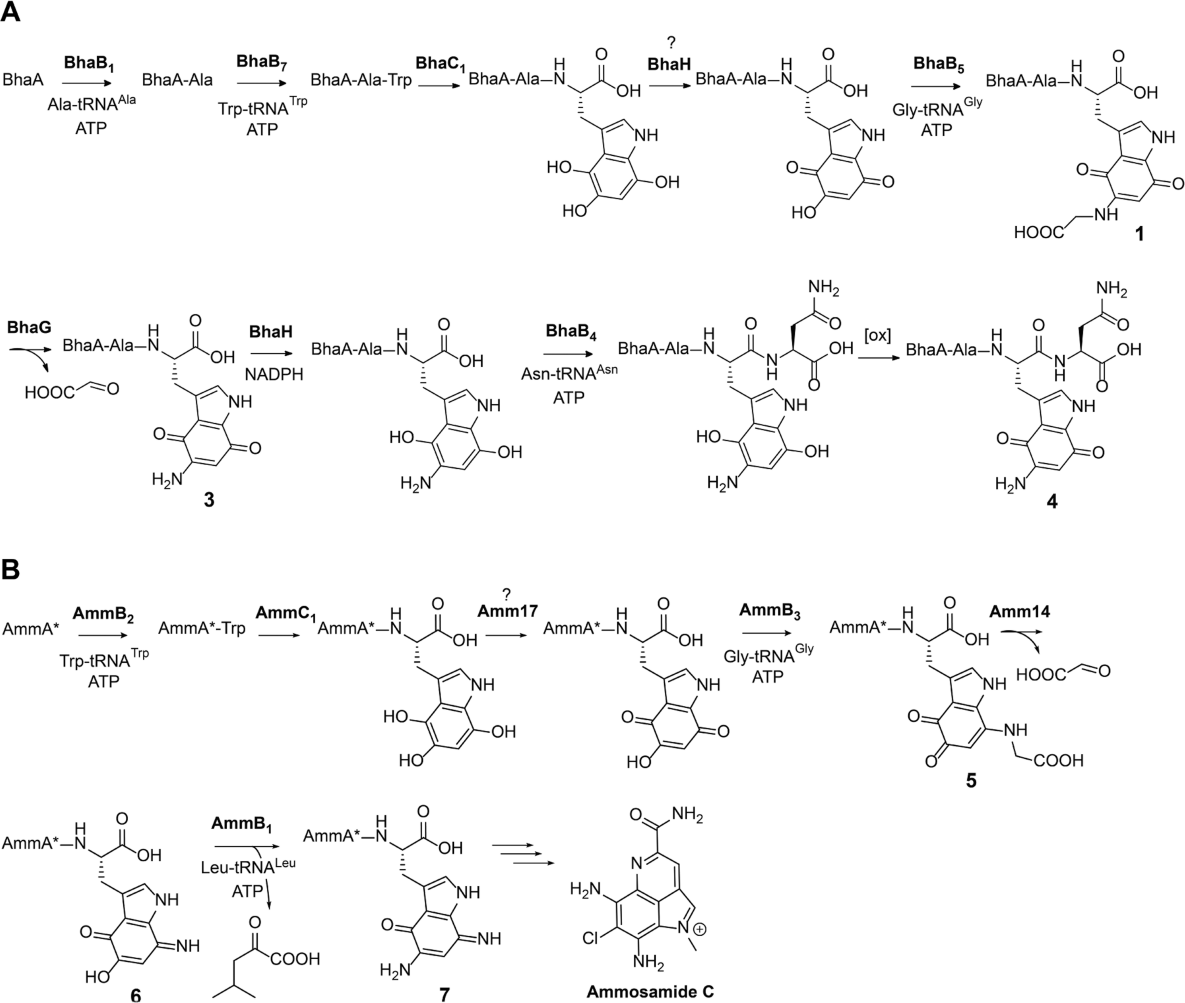
Updated functions of biosynthetic enzymes from the *bha* and *amm* BGCs. (A) Pathway encoded by the *bha* BGC based on the data in this and previous studies. (B) Pathway toward ammosamide C encoded by the *amm* BGC based on the data in this and previous studies.

We expressed BhaH as an N-terminal His_6_-tagged fusion protein that copurified with FMN. The purified enzyme reduced the standard substrate menadione in the presence of NADPH (Figure S8). We next investigated whether aminoquinone **3** would be a substrate for Asn conjugation by BhaB_4_ in the presence of BhaH. We previously observed rapid oxidation of the hydroquinones in the *bha* pathway under ambient conditions,^4^ and because BhaH was anticipated to generate such a hydroquinone from intermediate **3**, we performed the asparagine condensation reaction catalyzed by BhaB_4_ under anaerobic conditions in the presence of BhaH and NADPH. Indeed, we observed the complete formation of the +114 product, as shown by MALDI-TOF MS and high-resolution MS/MS analysis, whereas no product formation was observed when BhaH or NADPH was omitted from the reaction mixture (Figures S7A and S8C). Having identified conditions for full conversion, we then used the anaerobic conditions with isotopically labeled L-^15^N_2_–Asn for the *in vitro* assay with BhaB_4_ and BhaH. Using LC-MS analysis, the isotopically labeled asparagine was incorporated in the product (M+2 Da; Figure S9). Thus, the substrate for BhaB_4_ is likely the reduced hydroquinone form of **3**, which is then condensed with Asn at the C-terminal carboxylate in an Asn-tRNA^Asn^- and ATP-dependent manner (Figure 4A). This product oxidizes upon exposure to oxygen during sample preparation for high-resolution MS/MS analysis providing peptide **4**. The reason why peptide **3** might need to be reduced by BhaH for BhaB_4_ activity is not entirely clear. One possibility is that it prevents reaction at the vinylogous carboxylate that is present at C7 in the imine tautomer of **3**; such a vinylogous carboxylate at C5 is the site of condensation by BhaB_5_ (Figure 1B).^4^

### Different Glycine Adduct Is Obtained in the *Amm* Pathway Than in the *Bha* Pathway

In a previous study, we demonstrated using NMR spectroscopy that in the *bha* pathway, a glycine moiety is added by BhaB_5_ to the Trp core at position 5 of the indole moiety to generate **1** (Figure 1D).^4^ In the same study, it was also shown by MS that AmmB_3_ adds a Gly to the oxidized indole of a C-terminal Trp in its substrate (AmmA*-Trp), but the product was not characterized by NMR spectroscopy. Since the final product of the *amm* BGC is known (ammosamide C, Figure 1D), two amine groups need to be installed onto the indole. Although it appeared most likely that AmmB_3_ would also add Gly to C5, like BhaB_5_, we could not rule out that tautomerization involving positions 5 and 7 of the quinone in the substrate would result in activation of the latter for the condensation step.^4^ Hence, in the current work, we coexpressed His_6_-AmmA*-Trp, AmmC_1_, and AmmB_3_ in *E. coli* in a large scale (over 30 L) and purified the product peptide **5** for NMR characterization. After trypsin digestion, the C-terminal fragment containing the modified IAPLALAw (w = Gly-modified Trp) sequence was obtained and analyzed by multidimensional NMR spectroscopy. The data revealed that the site of glycine addition by AmmB_3_ is different from that observed for BhaB_5_. ^1^H–^13^C HMBC revealed correlations from the NH proton of the glycine adduct (δ 7.82 ppm) to the indole carbon at C8 (δ 131.90 ppm; for carbon numbering, see Figure 1D), indicating that the glycine addition occurred at C7 (intermediate **5**, Figure 4B). Nuclear overhauser effect spectroscopy (NOESY) data confirmed this assignment by the observation of NOE cross-peaks from the NH proton (δ 7.82 ppm) of the appended glycine to the NH proton (δ 11.92 ppm) of the indole moiety as well as from the CH_2_ protons (δ 4.10 ppm) of the appended glycine to the indole CH proton at C6 (δ 5.00 ppm) (Figure S10). As observed previously for the C5 adduct in the *bha* pathway,^4^ the glycine adduct at C7 was unstable and decomposed over time during our NMR measurements.

### Further Insights into the Flavin-Dependent Indole Trihydroxylase BhaC_1_

**Figure 5:**
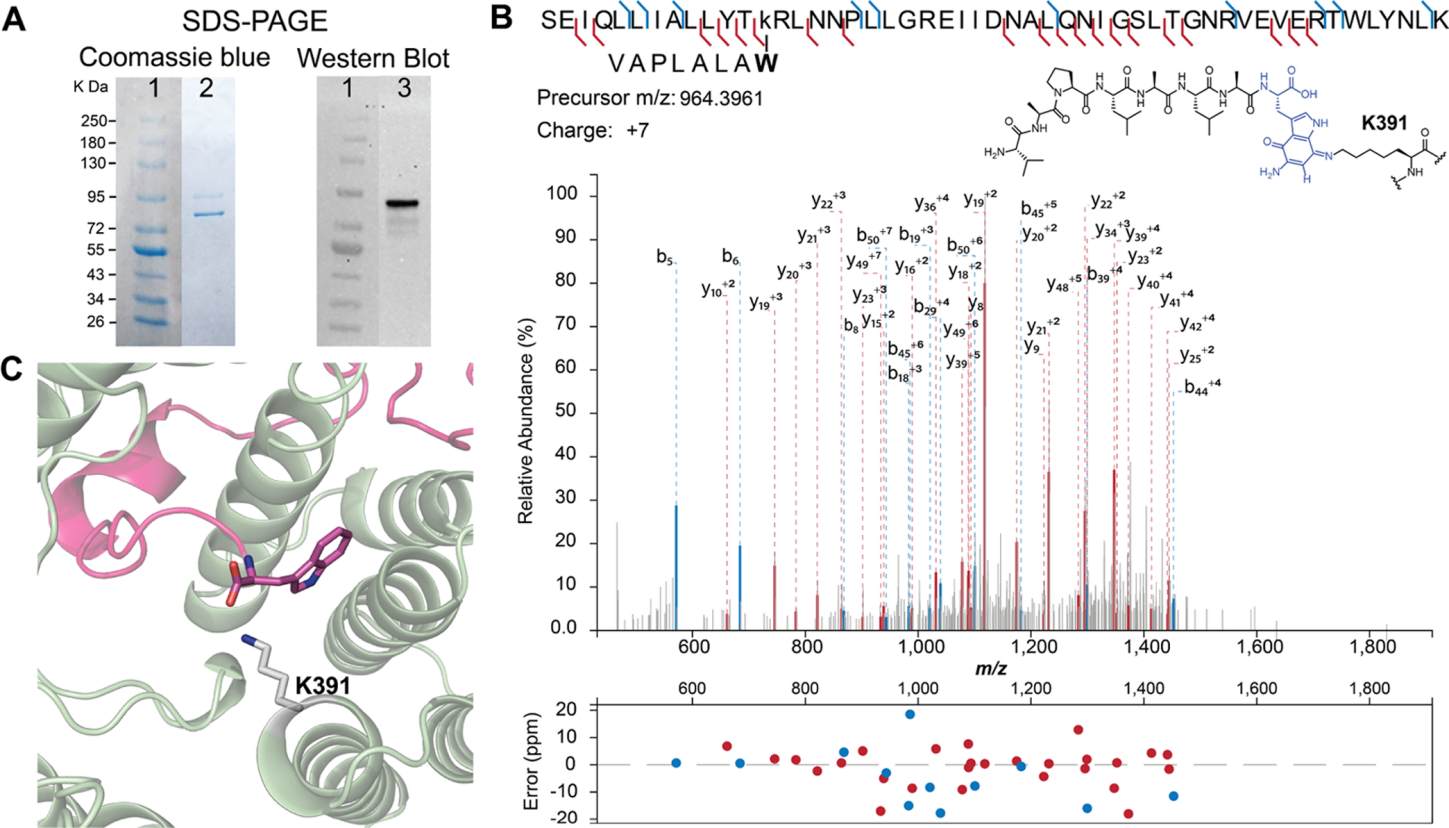
(A) SDS-PAGE analysis of the coexpression product obtained from non-His-tagged BhaC_1_ and non-His-tagged BhaB_5_ coexpressed with His-tagged BhaA-AW. The Coomassie blue stained gel (lane 2) shows two high molecular weight bands around ∼90 kDa and ∼80 kDa. Western blot analysis (lane 3) showed that the top band contains a histidine tag, likely corresponding to a covalent cross-link of intermediate **3** to BhaC_1_. (B) LC-MSMS data on a LysC-digest of the upper band. The fragment shown consists of a LysC-fragment of BhaC_1_ cross-linked to the C-terminal fragment of modified Bha-AW. The cross-link is drawn at C7 of the indole, but it could also be at C4. For additional tandem MS data supporting the assignment, see Figure S11. (C) AlphaFold Multimer prediction of the binding of BhaC1 (colored sage) to BhaA_AW (pink) positioning the C-terminal Trp in proximity to Lys391.

The observation that AmmB_3_ introduces an amino group from Gly-tRNA at C7 of the hydroxyquinone to give **5**, whereas BhaB_5_ catalyzes the same reaction at C5 to give **1** (Figure 4) may also explain why in the absence of the glycine oxidases AmmG/BhaG the major observed products formed by coexpression of the respective enzymes in *E. coli* are different. For the Bha pathway, the major product is the glycine adduct **1** at C5 (Figure S2), but for the Amm pathway, it is the imine **2’** at C7 (or its tautomer **2''**, Figure S10E).^4^ Apparently, the decarboxylation of the glycine adduct at C7 is easier than that at C5. The different reactions catalyzed by BhaB_5_ and AmmB_3_ may also explain one additional observation. When we coexpressed His_6_-BhaA-Ala-Trp with BhaC_1_ and BhaB_5_, we consistently observed a new band by sodium dodecyl sulfate polyacrylamide gel electrophoresis that runs at a slightly higher molecular weight than BhaC_1_ (Figure 5A). Such a band was not observed when BhaB_5_ was omitted, nor was it observed for the equivalent experiment with His_6_-AmmA*-Trp, AmmC_1_, and AmmB_3_. The protein that is associated with this band contains a His-tag as shown by Western blot analysis (Figure 5A). We isolated the band from the gel, digested the protein with LysC, and analyzed the digest by mass spectrometry. Fragments corresponding to sequences of both BhaC_1_ and BhaA-Ala-Trp were observed, suggesting that the band corresponds to a covalently cross-linked protein. Indeed, a fragment was observed that links a peptide spanning residues 382–432 of BhaC_1_ to the C-terminus of modified BhaA-Trp. Tandem MS analysis suggested that the linkage is between Lys391 and the aminoquinone of modified BhaA (Figures 5B and S11). The reaction catalyzed by BhaC_1_, trihydroxylation of the indole of Trp, is unique in biochemistry and bioinformatics did not predict this tetratrico repeat domain-containing protein to be a flavoprotein.^4,16^ Because the protein does not contain any canonical flavin binding sites, we previously used AlphaFold Multimer^18^ to predict the interaction between the substrate peptide (BhaA-Ala-Trp) and BhaC_1_,^16^ and thereby the putative position of the active site of BhaC_1_. The observed covalent interaction between Lys391 of BhaC_1_ and the oxidized indole of BhaA-Ala-Trp provides further support for the postulated active site position as Lys391 was predicted to interact with the C-terminal carboxylate of the BhaA-Ala-Trp peptide (Figure 5C). The high reactivity of the BhaB_5_ product was reported previously, and apparently, it results in cross-linking to BhaC_1_ when the amino group is at C5 but not cross-linking to AmmC_1_ when the amino group is at C7. Importantly, the observed adduct is consistent with the mechanism of substrate engagement predicted by AlphaFold Multimer.

### AmmB_1_ Is a Leu-tRNA^Leu^-Dependent PEARL Enzyme

For the *bha* system, we were able to identify the next step after Gly addition by coexpression with the Gly oxidase BhaG. We therefore examined whether the same might be true for the *amm* system. Given that the glycine adduct **5** formed by AmmB_3_ is an isomer of **1** and that the gene encoded by Amm14 (Figure 1A) is a putative glycine oxidase homologue of BhaG, we first coexpressed Amm14 with AmmA*-Trp, AmmC_1_, and AmmB_3_. However, we were not able to oxidize the glycine adduct in our coexpressions. Assuming that this might be a problem of expression of active Amm14, we investigated whether the glycine oxidase in the *bha* cluster (26% sequence identity to Amm14) could be used to catalyze 7-aminoquinone formation in the peptide. After including BhaG in the coexpression system described above, full conversion to the corresponding aminoquinone **6** was indeed observed (Figures S12 and 4B). High-resolution MS/MS analysis showed that the modification was localized at Trp. Thus, BhaG can oxidize Gly conjugated at either C5 or C7 of the oxidized indole of a C-terminal Trp.

**Figure 6:**
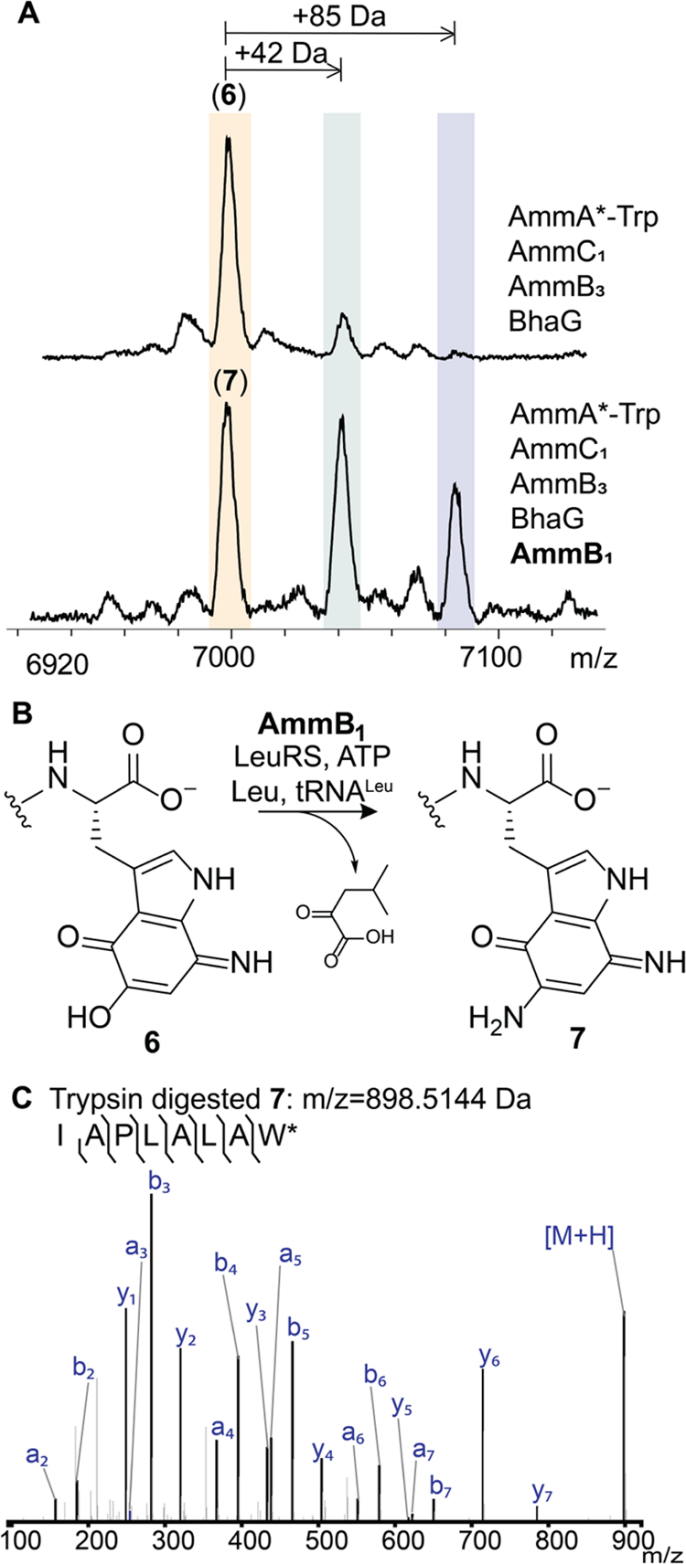
AmmB_1_ donates an amino group to the indole of intermediate **6**. (A) Inclusion of AmmB_1_ in the coexpression system that produces **6** results in the formation of three new products (Figure S13). (B) The reaction catalyzed by AmmB_1_. (C) ESI-MS/MS analysis of the C-terminal fragment upon trypsin digestion of peptide **7** (calculated *m*/*z* = 898.5144, observed *m*/*z* = 898.5181).

We next investigated whether the formation of the aminoquinone in the *amm* pathway would unlock a new biosynthetic step en route toward the formation of the ammosamide core, similar to the assignment of BhaB_4_ as an Asn-tRNA ligase discussed above. Therefore, we coexpressed AmmA*-Trp with the Bha homologues of AmmC_1_ (BhaC_1_) and Amm14 (BhaG), AmmB_3_, and the two remaining PEARLs in the *amm* cluster, AmmB_1_ and AmmB_4_, in separate cultures. After purification of the full-length peptide and MALDI-TOF MS analysis, only coexpression with AmmB_1_ showed a distinct MALDI-TOF MS profile that showed the seemingly unreacted aminoquinone **6** as well as two additional peaks corresponding to an increase in mass of 42 and 85 Da compared to **6** (Figure 6A). High-resolution MS/MS of the trypsin-digested mixture revealed that the apparently unmodified aminoquinone corresponded to a product that had decreased in mass by 1 Da from the aminoquinone intermediate **6** (i.e., product **7**, Figure 6B), and that the +42 and +85 Da products corresponded to acetylated **6** and bisacetylated reduced hydroquinone **7** as shown in Figure S13A. The most likely explanation is that product **7** is formed by addition of an amino acid by AmmB_1_ and subsequent conversion to the diaminoquinone (Figure 6B). In *E. coli*, both starting peptide **6** and peptide **7** (or its initially formed hydroquinone derivative) are then acetylated by an unknown enzyme.

To first identify the cryptic amino acid adduct and avoid acetylation, an *in vitro* reaction was conducted that included AmmB_1_, a mixture of *E. coli* AARS and tRNAs as well as purified aminoquinone intermediate **6**. Using a mixture of all 20 L-amino acids labeled with ^15^N, we observed full incorporation of one ^15^N into diaminoquinone **7** (Figure S14). We tested our initial hypothesis that predicted the use of Gly-tRNA^Gly^ as the second amino group donor of the indole core. However, the corresponding *in vitro* reaction did not yield any product. Next, we performed *in vitro* reactions with the remaining amino acids split into three groups (Figure S14B) and found that the group containing leucine, isoleucine, valine, alanine, and proline gave the previously observed −1 Da product **7**. Individual amino acids from this group were then tested and only Leu reproduced the formation of the corresponding diaminoquinone core (Figure S14C). Thus, tRNA^Leu^ and N-terminally His_6_-tagged LeuRS from *E. coli* were expressed and purified. Using L-^15^N-leucine, LeuRS, and tRNA^Leu^, we observed the formation of the diaminoquinone product **7** with an increase in mass by 1 Da (Figure S14B). While the diaminoquinone product is observed in this reaction, we were not able to observe the anticipated leucine-adduct intermediate. This finding could indicate that AmmB_1_ has dual activity, first catalyzing the appendage of the leucine moiety to the Trp core and also the conversion to the observed product **7**. Two different pathways can be envisioned for the latter process that would produce either isovaleraldehyde or 4-methyl-2-oxovaleric acid from Leu (Figure S15A). To test these hypotheses, we incubated the *in vitro* reaction products with 2,4-dinitrophenylhydrazine. As shown in Figure S15A, we observed the formation of the hydrazone adduct of 4-methyl-2-oxovaleric acid. This conclusion was confirmed by utilizing an authentic standard as well as several isotopically labeled l-leucine derivatives that all supported a hydrolytic mechanism, Figures S15B and S15C.

### Possible Origin of the PEARL Enzyme Class

The discovery in 2015 that class I lanthipeptide dehydratases (LanBs) use glutamyl-tRNA to dehydrate Ser and Thr residues through transesterification to form a glutamyl ester followed by glutamate elimination was surprising as no such biochemical transformations had been reported previously.^15^ Furthermore, the X-ray structure of a representative enzyme (NisB, UniProtKB P20103, PDB ID 4WD9) did not show any obvious structural homology with characterized enzymes to provide insights into where this activity may have evolved from. A subset of LanB enzymes, called split LanBs, are encoded as two polypeptides, with one subunit catalyzing the glutamylation reaction and the second subunit catalyzing glutamate elimination.^19−21^ The discovery that the related PEARL enzymes that contain a glutamylation-like domain but not the glutamate elimination domain added amino acids to the C-termini of peptides in an aminoacyl-tRNA and ATP-dependent process was again surprising given that the lanthipeptide dehydratases do not utilize ATP. Here, we used bioinformatic analysis as well as structure prediction by AlphaFold^22^ to try and answer some of these outstanding questions.

**Figure 7:**
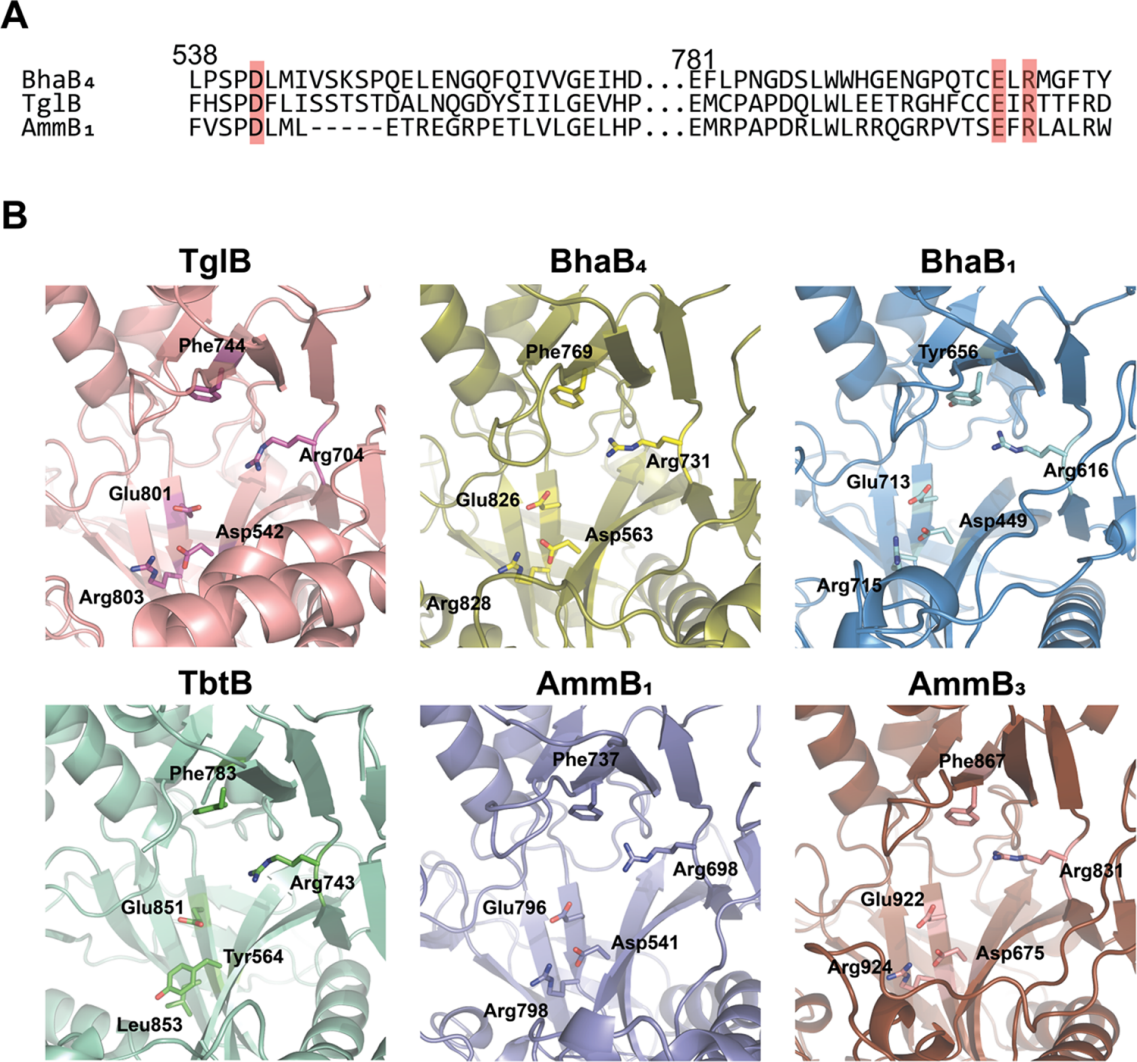
Conserved residues in PEARL enzymes versus TbtB, a member of the split LanB enzyme class. (A) Multiple Sequence Comparison by Log-Expectation (MUSCLE) alignment of three PEARL enzymes TglB, BhaB_4_, and AmmB_1_. Conserved residues (Asp542, Glu801, and Arg803; TglB numbering) located in the putative ATP-binding pocket are highlighted in red. (B) AlphaFold-predicted structures of the PEARL TglB (pink) and split LanB TbtB (green) in comparison with two representative examples of the PEARL enzymes in the *bha* and *amm* clusters. The residues corresponding to Arg743, Phe783, and Glu851 in TbtB are conserved in all structures, but Asp542 and Arg803 of TglB are only conserved in the PEARLs and substituted by Tyr and Leu in split LanB enzymes.

We constructed AlphaFold models of the PEARLs from the *bha* and *amm* clusters and used these models to gain insights into the putative catalytic region (Figure 7). The predicted PEARL structures revealed similar overall three-dimensional structures (e.g., Figure S16) with an apparent active site pocket resembling that of the crystallographically characterized split LanB, TbtB (UniProtKB D6Y502, PDB ID 6EC8), that catalyzes tRNA-dependent Ser glutamylation.^23^ In the TbtB structure, this pocket is occupied by a nonhydrolyzable substrate mimic of a 3′-glutamylated adenosine that interacts with several residues through H-bonding and π–π stacking interactions as shown in Figure S17. Two of the residues interacting with the adenine of the nonhydrolyzable mimic (Arg743 and Phe783 in TbtB) are also conserved in the PEARLs, as is a glutamic acid residue (Glu851, Figure 7). We hypothesize that in PEARLs, this region may alternately bind the adenosine of ATP and the 3′ terminus of a charged tRNA molecule, with the adenine moiety of both substrates interacting with the conserved Phe and Arg.

We compared the residues in this region of several AlphaFold-predicted PEARL structures as well as TglB (UniProtKB A0A8T8BZ30), a PEARL found in the thiaglutamate BGC that has been investigated by site-directed mutageneis.^2^ Two residues are structurally conserved in the predicted PEARL structures that are not present in TbtB, Asp542, and Arg803 (TglB numbering; Figure 7). Mutagenesis studies of these residues in TglB affected the ATP-dependent phosphorylation step that activates the C-terminus of the precursor peptide required for amino acid condensation via aminoacyl-tRNA transfer (Figure 1A).^2^ Thus, these prior mutagenesis studies on TglB are consistent with ATP binding in the same pocket as the nonhydrolyzable mimic of aminoacyl-tRNA.

Next, we searched for *bha* or *amm* sequence homologues using the Basic Local Alignment Search Tool (BLAST)^24^ with AmmB_4_ as query. While the proteins with the highest similarity scores were other PEARLs, split LanBs, and full-length LanBs, we also retrieved considerably shorter protein sequences (approximately half the size of PEARLs) with over 30% sequence identity (Figure S18). Intrigued by this result, we submitted these sequences for AlphaFold analysis and the resulting structures to the Dali Server. The results of this 3D-based search indicated that the N-terminal portion of the shorter PEARL sequence resembled previously structurally characterized glutathionylspermidine synthase and glutathione synthetase, a founding member of the ATP-GRASP family^25^ (Figure S19). Like the PEARLs, these two enzymes catalyze condensation reactions at the C-terminus of a peptide in an ATP-dependent manner involving first phosphorylation of the C-terminal carboxylate, followed by attack of a nucleophilic amine onto the activated acylphosphate.

A comparison between glutathione (GSH) synthase and PEARL sequences and predicted structures revealed a similar constellation of residues Glu281, Asp273, Arg210, and Asp208 in GSH synthase with the corresponding residues in the PEARLs (e.g., Glu564, Asp542, Arg803, and Glu801 in TglB, Figure S19). In the structure of GSH synthase bound to ADP (PDB 1GSA),^26,27^ Glu281 along with Asn283 mediates binding of two Mg^2+^ ions that coordinate to the β and α phosphates of ADP, and Asp273 binds to the Mg^2+^ that interacts with the α-phosphate. Arg210 and Asp208 are near the putative γ phosphate site (substituted by a sulfate group in the crystal structure) and the hydroxyl group in the ribose ring of ATP. In TglB, Glu564 was shown not to be essential for catalysis and Asn283 is replaced by a His residue that is highly conserved in PEARLs. These observations suggest that during the evolution of ATP-GRASP enzymes to PEARLs, some of the residues that interacted with ATP in the former were changed, such that the latter could accommodate aminoacyl-tRNA in the same binding site. The split LanBs may have evolved from PEARLs by recruitment of the elimination domain that has evolutionary links to a number of other proteins (thiopeptide cyclases, LsrG epimerase),^28,29^ and the LanB lanthipeptide dehydratases likely arose from a subsequent gene fusion of the tRNA-dependent domain and the elimination domain.

## Conclusions

After the previous unexpected discovery that PEARLs add glycine from Gly-tRNA to hydroxyquinones derived from Trp during the biosynthesis of pyrroloiminoquinone natural products,^4^ we proposed a chemically plausible route to ammosamide C that was efficient in terms of step-count (Figure S1) but did not use all of the biosynthetic genes in the BGC. In this study, we demonstrate the activity of some of these enzymes that allowed the assignment of new enzymatic activities for two previously uncharacterized PEARLs in the *bha* and *amm* BGCs. Our data show that the amine groups in ammosamide do not all originate from glycine as we had anticipated nor are they introduced using similar chemistry. Instead, for ammosamide, we show that the nitrogen at C7 of the former indole is derived from glycine but the amine at C5 is derived from leucine. Furthermore, the mechanisms of cleavage of the N–Cα bonds of these two amino acids during the amino transfer process is quite different. For the introduction of the amine at position 7, a glycine oxidase is required to efficiently convert the initial glycine condensation product to the aminoquinone product, a reaction that produces glyoxylate. Conversely, for the introduction of the amino group at C5, a tautomerization and hydrolysis process appears to be operational (Figure S15).

The glycine oxidase enzyme is encoded in both the *amm* and *bha* BGCs, and its activity unlocked new PEARL reactions in both pathways. In the *amm* system, previous attempts to reconstitute the pathway beyond the glycine addition step catalyzed by AmmB_3_ stalled at intermediate **2’** (or its tautomer, Figure S10E), which we now consider a dead-end product formed by nonenzymatic decarboxylation when the glycine oxidase is absent. In the presence of the glycine oxidase, the aminoquinone intermediate was efficiently formed, which allowed the function of AmmB_1_ to be determined (Figure 4). Similarly, without the glycine oxidase, the *bha* pathway produced a mixture of products **1**–**3**, of which only the minor product (**3**) is a competent substrate for BhaB_4_. In addition to showing that the glycine oxidases are important for both pathways, our study revealed another important enzyme that assures the formation of the correct oxidation state of an intermediate for subsequent enzyme catalysis. The data show that the quinone reductase BhaH (and presumably its homologue Amm17 in the *amm* BGC) is required for BhaB_4_ activity by reducing the initially formed aminoquinone to the hydroquinone form. We suggest that the quinone reductases BhaH and Amm17 are involved in several steps of the pathway to reversibly interconvert quinone and hydroquinone forms of the former Trp indole. For instance, the initial BhaC_1_ and AmmC_1_ products are hydroxylated indoles (Figure 4), but for the PEARL chemistry catalyzed by AmmB_3_ and BhaB_5_, a vinylogous carboxylate is required for ATP-GRASP-like phosphorylation, followed by an addition–elimination sequence with a tetrahedral intermediate. We suggest that the quinone reductases may oxidize the BhaC_1_/AmmC_1_ product for use by the PEARL enzymes (Figure 4). Conversely, for the chlorinase Amm3 involved in ammosamide C biosynthesis,^7^ for which we and others have not yet been able to identify the exact substrate, a nucleophilic reduced oxidation state is likely required.

The new data in the current work also indicate that whereas in previous studies the addition of Trp to a highly conserved peptide and its subsequent trihydroxylation were shown to be conserved between the *amm* and *bha* pathways,^4^ the subsequent steps diverge. AmmB_3_ and BhaB_5_ both add Gly from Gly-tRNA but the regiochemistry is different with AmmB_3_ adding Gly at C7 and BhaB_5_ at C5 of the indole. Similarly, the step after the glycine oxidase oxidation of the Gly differs. In the *amm* pathway, nitrogen from Leu-tRNA is incorporated into the quinone by AmmB_1_, whereas Asn-tRNA is added to the C-terminal carboxylate by BhaB_4_.

In this study, we identified the activities of two PEARLs. BhaB_4_ added Asn to the C-terminus of the peptide intermediate and AmmB_1_ added Leu to the oxidized aminoquinone core although the initial adduct was not detected and the addition is inferred from the isotope incorporation studies. Like all previously characterized PEARLs, these two activities were dependent on both ATP and the presence of the corresponding tRNA^Asn^ and tRNA^Leu^. Also, similar to previous studies, these enzymes worked with tRNA sequences from *E. coli* even though the enzymes originate from different bacterial phyla that use different tRNA sequences, suggesting that it is mostly the amino acid that confers substrate specificity. Three PEARLs in the *bha* cluster remain to be assigned, as well as one PEARL in the *amm* cluster. Given the surprises that the biosynthetic pathways to pyrroloiminoquinones have provided thus far, we consider it perilous to propose a possible pathway from the currently accessed last intermediate to ammosamide C. Although the functional assignment of BhaB_4_ provides new information on the possible structure of the cryptic *bha* BGC, the genes of unassigned function include three proteins containing tetraticopeptide repeat domains, a methyltransferase, and a hypothetical protein in addition to the three remaining PEARLs, preventing prediction of its mature structure without further investigation.

Ever since the mechanism of class I lanthipeptide dehydratases was shown to require glutamyl-tRNA, the evolutionary origin of these enzymes has remained enigmatic. Through the use of structure-predicting tools like AlphaFold and bioinformatic analysis, we have gained the first insights into both the three-dimensional features that may be important for PEARL catalysis and their potential evolutionary path. We suggest that PEARLs evolved from ATP-Grasp enzyme family members by recruiting a specialized nucleophile (aminoacyl-tRNA) to react with an activated acylphosphate substrate. It is possible that the recruitment of this new substrate was facilitated by the similarities in structure between ATP and the conserved adenosine at the 3′ end of aminoacylated tRNA. Additional outstanding questions include the molecular basis for the aminoacyl-tRNA specificities of PEARLs as well as the recognition of their peptide substrates.

## Supporting Information Available

Experimental procedures, Figures S1–S19 showing spectroscopic data and Table S1 with gene sequences (PDF)

## References

[ref1] Ting C. P.; Funk M. A.; Halaby S. L.; Zhang Z.; Gonen T.; van der Donk W. A. Use of a scaffold peptide in the biosynthesis of amino acid-derived natural products. Science 2019, 365, 280–284. 10.1126/science.aau6232. 31320540 PMC6686864

[ref2] Zhang Z.; van der Donk W. A. Nonribosomal peptide extension by a peptide amino-acyl tRNA ligase. J. Am. Chem. Soc. 2019, 141, 19625–19633. 10.1021/jacs.9b07111. 31751505 PMC6927032

[ref3] Yu Y.; van der Donk W. A. Biosynthesis of 3-thia-α-amino acids on a carrier peptide. Proc. Natl. Acad. Sci. U.S.A. 2022, 119, e2205285119 10.1073/pnas.2205285119. 35787182 PMC9303977

[ref4] Daniels P. N.; Lee H.; Splain R. A.; Ting C. P.; Zhu L.; Zhao X.; Moore B. S.; van der Donk W. A. A biosynthetic pathway to aromatic amines that uses glycyl-tRNA as nitrogen donor. Nat. Chem. 2022, 14, 71–77. 10.1038/s41557-021-00802-2. 34725492 PMC8758506

[ref5] Hughes C. C.; MacMillan J. B.; Gaudencio S. P.; Jensen P. R.; Fenical W. The ammosamides: structures of cell cycle modulators from a marine-derived *Streptomyces* species. Angew. Chem., Int. Ed. 2009, 48, 725–727. 10.1002/anie.200804890. PMC2819817 19090514

[ref6] Luo J.; Yang D.; Hindra; Adhikari A.; Dong L.-B.; Ye F.; Yan X.; Rader C.; Shen B. Discovery of ammosesters by mining the *Streptomyces uncialis* DCA2648 genome revealing new insight into ammosamide biosynthesis. J. Ind. Microbiol. Biotechnol. 2021, 48, kuab027 10.1093/jimb/kuab027. 33982054 PMC8210675

[ref7] Jordan P. A.; Moore B. S. Biosynthetic pathway connects cryptic ribosomally synthesized posttranslationally modified peptide genes with pyrroloquinoline alkaloids. Cell Chem. Biol. 2016, 23, 1504–1514. 10.1016/j.chembiol.2016.10.009. 27866908 PMC5182094

[ref8] Hughes C. C.; MacMillan J. B.; Gaudencio S. P.; Fenical W.; La Clair J. J. Ammosamides A and B target myosin. Angew. Chem., Int. Ed. 2009, 48, 728–732. 10.1002/anie.200804107. PMC2820877 19097126

[ref9] Hu J. F.; Fan H.; Xiong J.; Wu S. B. Discorhabdins and pyrroloiminoquinone-related alkaloids. Chem. Rev. 2011, 111, 5465–5491. 10.1021/cr100435g. 21688850

[ref10] Miyanaga A.; Janso J. E.; McDonald L.; He M.; Liu H.; Barbieri L.; Eustaquio A. S.; Fielding E. N.; Carter G. T.; Jensen P. R.; et al. Discovery and assembly-line biosynthesis of the lymphostin pyrroloquinoline alkaloid family of mTOR inhibitors in *Salinispora* bacteria. J. Am. Chem. Soc. 2011, 133, 13311–13313. 10.1021/ja205655w. 21815669 PMC3161154

[ref11] Reddy P. V. N.; Jensen K. C.; Mesecar A. D.; Fanwick P. E.; Cushman M. Design, synthesis, and biological evaluation of potent quinoline and pyrroloquinoline ammosamide analogues as inhibitors of quinone reductase 2. J. Med. Chem. 2012, 55, 367–377. 10.1021/jm201251c. 22206487 PMC3262027

[ref12] Colosimo D. A.; MacMillan J. B. Ammosamides unveil novel biosynthetic machinery. Cell Chem. Biol. 2016, 23, 1444–1446. 10.1016/j.chembiol.2016.12.001. 28009976

[ref13] Lin S.; McCauley E. P.; Lorig-Roach N.; Tenney K.; Naphen C. N.; Yang A. M.; Johnson T. A.; Hernadez T.; Rattan R.; Valeriote F. A.; Crews P. Another look at pyrroloiminoquinone alkaloids-perspectives on their therapeutic potential from known structures and semisynthetic analogues. Mar. Drugs 2017, 15, 98 10.3390/md15040098. 28353633 PMC5408244

[ref14] Reimer D.; Hughes C. C. Thiol-based probe for electrophilic natural products reveals that most of the ammosamides are artifacts. J. Nat. Prod. 2017, 80, 126–133. 10.1021/acs.jnatprod.6b00773. 28055208

[ref15] Ortega M. A.; Hao Y.; Zhang Q.; Walker M. C.; van der Donk W. A.; Nair S. K. Structure and mechanism of the tRNA-dependent lantibiotic dehydratase NisB. Nature 2015, 517, 509–512. 10.1038/nature13888. 25363770 PMC4430201

[ref16] Daniels P. N.; van der Donk W. A. Substrate specificity of the flavoenzyme BhaC(1) that converts a C-Terminal Trp to a hydroxyquinone. Biochemistry 2023, 62, 378–387. 10.1021/acs.biochem.2c00206. 35613706 PMC9850906

[ref17] Settembre E. C.; Dorrestein P. C.; Park J.-H.; Augustine A. M.; Begley T. P.; Ealick S. E. Structural and mechanistic studies on ThiO, a glycine oxidase essential for thiamin biosynthesis in *Bacillus subtilis*. Biochemistry 2003, 42, 2971–2981. 10.1021/bi026916v. 12627963

[ref18] Evans R.; O’Neill M.; Pritzel A.; Antropova N.; Senior A.; Green T.; Žídek A.; Bates R.; Blackwell S.; Yim J.; et al. Protein complex prediction with AlphaFold-Multimer. bioRxiv 2021, 2021-10 10.2021/10.04.463034.

[ref19] Hudson G. A.; Zhang Z.; Tietz J. I.; Mitchell D. A.; van der Donk W. A. In vitro biosynthesis of the core scaffold of the thiopeptide thiomuracin. J. Am. Chem. Soc. 2015, 137, 16012–16015. 10.1021/jacs.5b10194. 26675417 PMC4819586

[ref20] Zhang Z.; Hudson G. A.; Mahanta N.; Tietz J. I.; van der Donk W. A.; Mitchell D. A. Biosynthetic timing and substrate specificity for the thiopeptide thiomuracin. J. Am. Chem. Soc. 2016, 138, 15511–15514. 10.1021/jacs.6b08987. 27700071 PMC5148741

[ref21] Ozaki T.; Kurokawa Y.; Hayashi S.; Oku N.; Asamizu S.; Igarashi Y.; Onaka H. Insights into the biosynthesis of dehydroalanines in goadsporin. ChemBioChem. 2016, 17, 218–223. 10.1002/cbic.201500541. 26630235

[ref22] Jumper J.; Evans R.; Pritzel A.; Green T.; Figurnov M.; Ronneberger O.; Tunyasuvunakool K.; Bates R.; Žídek A.; Potapenko A.; et al. Highly accurate protein structure prediction with AlphaFold. Nature 2021, 596, 583–589. 10.1038/s41586-021-03819-2. 34265844 PMC8371605

[ref23] Bothwell I. R.; Cogan D. P.; Kim T.; Reinhardt C. J.; van der Donk W. A.; Nair S. K. Characterization of glutamyl-tRNA-dependent dehydratases using nonreactive substrate mimics. Proc. Natl. Acad. Sci. U.S.A. 2019, 116, 17245–17250. 10.1073/pnas.1905240116. 31409709 PMC6717260

[ref24] Altschul S. F.; Gish W.; Miller W.; Myers E. W.; Lipman D. J. Basic local alignment search tool. J. Mol. Biol. 1990, 215, 403–410. 10.1016/S0022-2836(05)80360-2. 2231712

[ref25] Iyer L. M.; Abhiman S.; Maxwell Burroughs A.; Aravind L. Amidoligases with ATP-grasp, glutamine synthetase-like and acetyltransferase-like domains: synthesis of novel metabolites and peptide modifications of proteins. Mol. Biosyst. 2009, 5, 1636–1660. 10.1039/b917682a. 20023723 PMC3268129

[ref26] Hara T.; Kato H.; Katsube Y.; Oda Ji. A pseudo-Michaelis quaternary complex in the reverse reaction of a ligase: structure of *Escherichia coli* B glutathione synthetase complexed with ADP, glutathione, and sulfate at 2.0 Å resolution. Biochemistry 1996, 35, 11967–11974. 10.1021/bi9605245. 8810901

[ref27] Galperin M. Y.; Koonin E. V. A diverse superfamily of enzymes with ATP-dependent carboxylate-amine/thiol ligase activity. Protein Sci. 1997, 6, 2639–2643. 10.1002/pro.5560061218. 9416615 PMC2143612

[ref28] Cogan D. P.; Hudson G. A.; Zhang Z.; Pogorelov T. V.; van der Donk W. A.; Mitchell D. A.; Nair S. K. Structural insights into enzymatic [4 + 2] aza-cycloaddition in thiopeptide antibiotic biosynthesis. Proc. Natl. Acad. Sci. U.S.A. 2017, 114, 12928–12933. 10.1073/pnas.1716035114. 29158402 PMC5724283

[ref29] Marques J. C.; Lamosa P.; Russell C.; Ventura R.; Maycock C.; Semmelhack M. F.; Miller S. T.; Xavier K. B. Processing the interspecies quorum-sensing signal autoinducer-2 (AI-2): characterization of phospho-(S)-4,5-dihydroxy-2,3-pentanedione isomerization by LsrG protein. J. Biol. Chem. 2011, 286, 18331–18343. 10.1074/jbc.M111.230227. 21454635 PMC3093905

